# Clinicopathological Features and Survival Analysis in Molecular Subtypes of Muscle-Invasive Bladder Cancer

**DOI:** 10.3390/ijms24076610

**Published:** 2023-04-01

**Authors:** Francesca Sanguedolce, Ugo Giovanni Falagario, Magda Zanelli, Andrea Palicelli, Maurizio Zizzo, Stefano Ascani, Simona Tortorella, Vito Mancini, Angelo Cormio, Giuseppe Carrieri, Luigi Cormio

**Affiliations:** 1 Pathology Unit, Policlinico Riuniti, University of Foggia, 71122 Foggia, Italy; 2 Department of Urology and Renal Transplantation, Policlinico Riuniti, University of Foggia, 71122 Foggia, Italy; 3 Pathology Unit, Azienda USL-IRCCS di Reggio Emilia, 42123 Reggio Emilia, Italy; 4 Surgical Oncology Unit, Azienda USL-IRCCS di Reggio Emilia, 42123 Reggio Emilia, Italy; 5 Pathology Unit, Azienda Ospedaliera Santa Maria di Terni, University of Perugia, 05100 Terni, Italy; 6 Urology Unit, Azienda Ospedaliero-Universitaria Ospedali Riuniti Di Ancona, Università Politecnica Delle Marche, Via Conca 71, 60126, Ancona, Italy; 7 Department of Urology, Bonomo Teaching Hospital, 76123 Andria, Italy

**Keywords:** bladder cancer, immunohistochemistry, molecular subtyping

## Abstract

Molecular subtyping of bladder cancer (BC) aims to capture the biological heterogeneity of this complex disease in order to provide better patient risk stratification. Immunohistochemical (IHC) markers are regarded as promising surrogates to classify BCs into luminal and basal subtypes in routine practice. We investigated the correlation between the molecular subclassification, assessed through IHC, and the conventional prognostic variables of a cohort of 93 muscle-invasive BCs (MIBCs), with a focus on the pattern of muscularis propria (MP) invasion, and evaluated their association with outcome. Basal, luminal, double-positive (DP), and double-negative (DN) phenotypes were identified according to the coordinate expression of 1 basal (CK5/6) and 2 luminal (CK20, GATA3) markers, and accounted for 33.3%, 32.3%, 3.2%, and 31.2% (Scheme #1) and 9.7%, 60.2%, 26.9%, and 3.2% (Scheme #2). There was a significant association between the pattern of MP invasion and the molecular subtypes according to Scheme #2, in that all 8 basal and DN cases, as well as 83% of DP cases, had a non-infiltrative invasion pattern. No consistent differences were observed in terms of OS and CSS between the molecular subtypes obtained through surrogate IHC markers. In keeping with previous studies, we report the correlation between the identification of BC subtypes and the presence of morphological prognostic factors, supporting the need for a comprehensive pathological evaluation, including clinicopathological and molecular parameters, in order to improve the diagnosis and management of MIBC.

## 1. Introduction

Bladder cancer (BC) is the second most common genitourinary malignancy [1], with urothelial carcinoma (UC) comprising 90% of all bladder tumors [2]. Muscle-invasive bladder cancer (MIBC) comprises nearly a third of all cases, with more than 30% recurrence and overall 5-year recurrence-free survival rates ranging from 58 to 81% [3].

Since conventional clinical and pathological prognostic/predictive factors proved to be insufficiently effective to provide accurate risk stratification of MIBC patients [4], comprehensive genomic analysis has been performed, resulting in several attempts to stratify BC on the basis of mRNA profiling, as proposed by the Cancer Genome Atlas (TCGA) consortium [5], the MD Anderson Cancer Center [6], the University of North Carolina [7], and Lund University [8]. Ultimately, a consensus classification has been developed in order to summarize these findings and provide a comprehensive subtyping scheme [9]. These classification systems exhibit significant overlaps, with over 90% of MIBCs showing either basal or luminal features. The latter has activated PPAR-γ and FGFR3 mutations, with enriched epithelial markers, and a good clinical prognosis, whereas basal tumors are associated with EGFR regulon activity, usually present at an advanced stage, and have the worst clinical prognosis [10]; nevertheless, they seem to be more sensitive to neoadjuvant cisplatin-based chemotherapy than luminal MIBCs [11,12]. The routine performance of molecular analysis in current practice is undermined by technical and economic issues; thus, immunohistochemical (IHC) surrogates for molecular profiling have been identified by validating the consistency between mRNA expression profiles and the expression of immunohistochemical markers [13,14].

The prognostic role of the pathological assessment of tumor invasion patterns has been extensively analyzed in some tumors, mostly oral squamous cell carcinoma. On the basis of data derived from meta-analyses, the presence of a non-cohesive pattern of invasion has been listed among the histological factors associated with a worse prognosis in the latest *WHO Classification of Head and Neck Tumors* (5th Ed.) [15]. Accordingly, a recent study has highlighted the association between molecular subtypes of BC and specific patterns of muscularis propria (MP) invasion [16]. The role of classical prognostic features in such molecular scenarios needs to be further elucidated, and a comprehensive evaluation for each BC patient, encompassing both clinical and histological markers and molecular subtypes, is advisable.

The aim of this study is to examine the correlation between the molecular subclassification, assessed through IHC, and the conventional prognostic variables of a cohort of MIBCs, with a focus on the pattern of MP invasion, and to evaluate their association with outcome.

## 2. Results

### 2.1. Patient Characteristics

Ninety-three patients diagnosed with MIBC were included in the present study. Among them, 81 (87.1%) underwent radical cystectomy (RC), and 12 (12.9%) received a transurethral resection of the bladder tumor (TURBT). The cohort consisted of 14 female (15.1%) and 79 male (84.9%) patients. The patients’ ages ranged from 69 to 78 years (median, 74 years). None of them received neoadjuvant chemotherapy or radiotherapy.

The clinical and morphological characteristics of the overall population are reported in Table 1. The majority of tumors lacked papillary morphology (90.1%), anaplastic features (81.5%), and concomitant CIS (79.0%). Tumor size ranged from approximately 3 to 5 cm (median, 4 cm), and they mostly exhibited a non-infiltrative pattern of MP invasion. Patients were diagnosed with AJCC stage IIIA (55.6%), stage IIIB (33.3%), and stage II (11.1%), in decreasing order. LVI and PNI were present in 48.1% and 38.3% cases, respectively.

### 2.2. Expression of CK5/6, CK7, CK20, CK34βE12, and GATA3 and Their Association with Clinicopathological Variables

Overall, the most frequently expressed markers were GATA3 (*n* = 81, 87.1%), CK7 (*n* = 76, 81.7%), CK34βE12 (*n* = 72, 77.4%), CK5/6 (*n* = 34, 36.6%), and CK20 (*n* = 33, 35.5%), in decreasing order. In RC samples (*n* = 81), no significant difference was noted between the expression of each marker and age, papillary morphology, anaplasia, concomitant CIS, tumor stage, nodal status, AJCC stage, LVI, and PNI (all *p*-values > 0.05).

There was a significant association between the expression of CK5/6 and GATA3 and male gender (*p* = 0.028 and *p* = 0.030, respectively). CK7 positivity was consistently associated with conventional UC (*p* = 0.046). MIBCs with a non-infiltrative pattern of MP invasion showed significantly higher levels of CK5/6 (*p* = 0.009), CK34βE12 (*p* = 0.046), and GATA3 (*p* = 0.021) expression. The results of our analysis are summarized in Table 2.

### 2.3. Stratification of MIBCs into Molecular Subtypes According to Surrogate IHC Markers and Their Association with Clinicopathological Variables

Using 2 schemes based on the coordinate expression of 1 basal (CK5/6) and 2 luminal markers (CK20 and GATA3), we were able to classify patients into basal and luminal subtypes comprising 33.3% and 32.3% (Scheme #1) and 9.7% and 60.2% (Scheme #2) of the cases, respectively. Furthermore, double-positive (DP) and double-negative (DN) cases were detected according to each scheme, accounting for 3 (3.2%) and 29 (31.2%) (Scheme #1) and 25 (26.9%) and 3 (3.2%) (Scheme#2), respectively.

There was no significant association between the molecular subtypes obtained through Scheme #1 and the clinicopathological features (all *p*-values > 0.05). On the other hand, molecular subtypes according to Scheme #2 were consistently associated with the pattern of MP invasion (*p* = 0.018) (Table 3 and Table 4).

**Figure 1:**
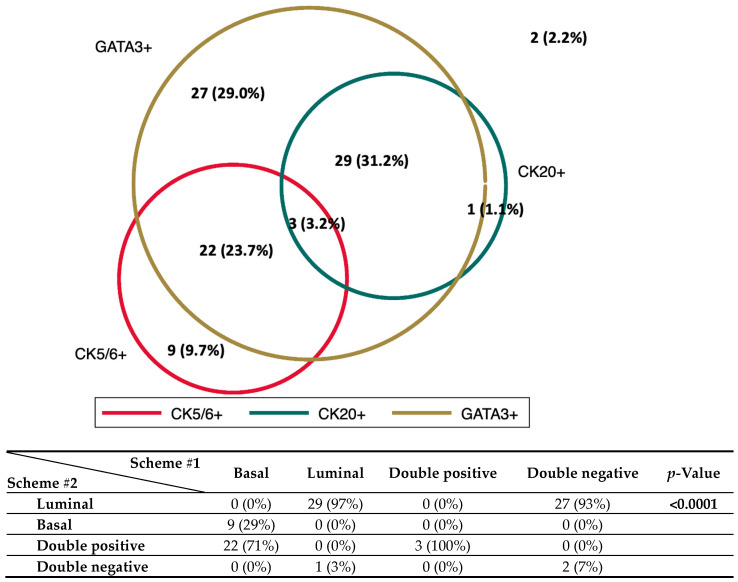
Venn diagram showing the expression of the 1 basal (CK5/6) and 2 luminal (CK20, GATA3) markers analyzed in this study. The relationships between the two schemes are summarized in the contingency table.

A Venn diagram showing the expression of CK5/6, CK20, and GATA3 is presented in Figure 1. The differences in the two classification schemes are mainly driven by the divergent expression of the luminal markers GATA3 and CK20, with 27 patients positive to GATA 3 but negative to CK20.

### 2.4. Survival Analysis

At the last available follow-up, 43 (46%) patients died from any cause, and 28 (30%) died from their disease.

**Figure 2:**
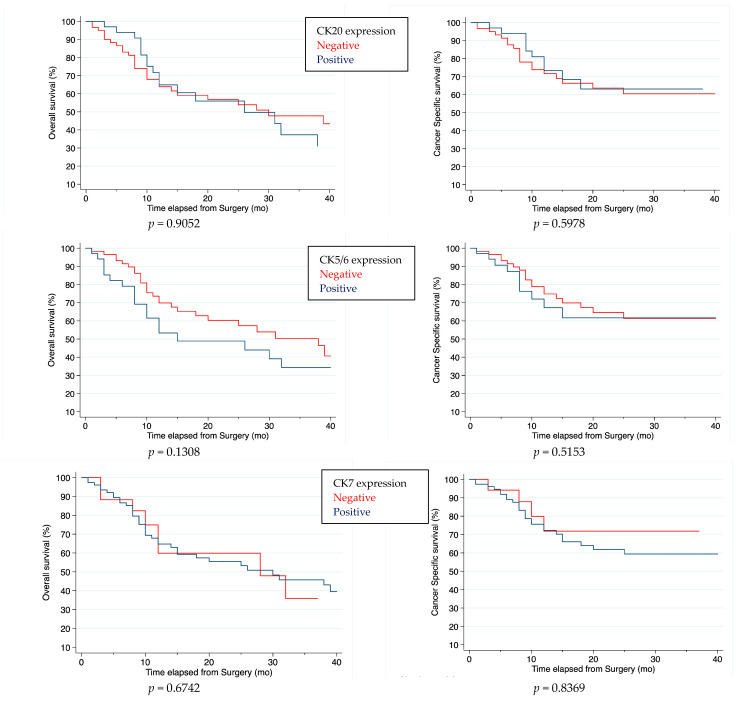

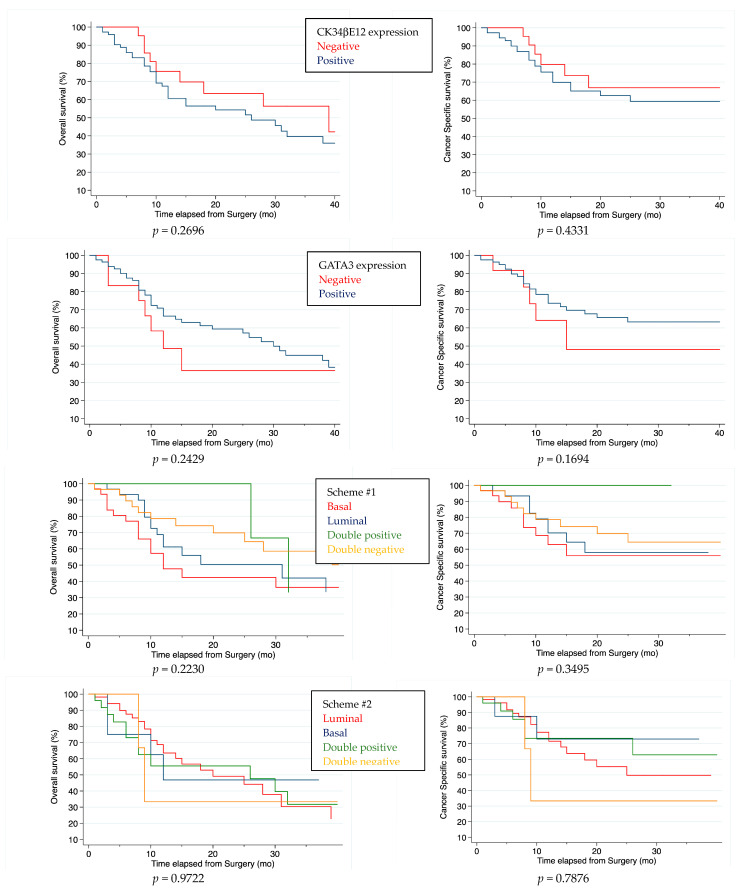
Kaplan–Meier analysis of single and combined marker expression.

Kaplan–Meier curves showed no differences in CSS and OS in the population stratified according to biomarker expression, both individually and combined into schemes (Figure 2).

## 3. Discussion

In this study, we have reported the immunohistochemical expression of a series of BC-associated markers, both individually and combined in molecular subtyping schemes, and its relationship to clinicopathological variables and survival in a cohort of 93 chemotherapy-naïve MIBCs.

Our analysis revealed that GATA3 was the most frequently expressed marker, being positive in as many as 81 cases (87.1%), whereas only 33 cases (35.5%) were CK20-positive. GATA3 has been recognized as a marker of urothelial lineage due to its commonly high expression rates in UC, and it is widely used in the distinction between primary and secondary tumors of the bladder [17]. Nevertheless, the range of GATA3 positivity in UC is definitely wide, spanning from less than 5% to 100% [14]. Consistently lower CK20 staining rates have been described in BCs (up to 70%) [18,19,20]. CK20 has been regarded as a luminal marker due to its expression in the most differentiated cells lying in the upper layers of normal urothelium, and CK20-positive tumors are enriched with luminal-type genes, such as FGFR3, FOXA1, and UPK2 [21]. A few potential basal markers have been assayed in previous studies, the most common being CK5/6, which stains basal and intermediate cells of normal urothelium. Kim et al. reported high expression of epithelial–mesenchymal transition and cell adhesion markers, as well as of TNF and MAPK signaling pathways [21].

The combined expression of one basal (CK5/6) and two luminal (GATA3 and CK20) markers was used to stratify MIBCs, according to two proposed schemes [21,22], into molecular subtypes. Studies based on transcriptome profiling of BC cohorts have resulted in the development of a few molecular classifications of MIBCs. Such subtypes were enriched with specific genetic changes, morphological features, and/or immune-related signatures, and showed distinct oncological behaviors and responses to neoadjuvant chemotherapy and immune checkpoint inhibitors. Recently, a consensus system by Kamoun et al. proposed to classify MIBCs into six groups, namely, Basal–Squamous, Luminal–Papillary, Luminal Non-Specified, Luminal Unstable, Stroma-rich, and Neuroendocrine-like [9]. Despite its advantages as a prognostic tool, applying an RNA-based molecular subtyping in current clinical practice is challenging since it is time- and cost-expensive and not universally available, hence the need to implement IHC as a surrogate method to identify specific markers at the protein level. A 2-antibody panel including CK5/6 and GATA3 has been assessed in previous studies as a surrogate classifier of molecular subtypes of BC, showing up to 91% concordance [23,24,25,26]. Other authors suggested the use of CK20 as a luminal marker instead of, or along with, GATA3, mostly due to the latter often being positive in MIBCs, irrespective of their molecular subtype [16,21]. Razzaghdoust et al. applied a CK5/6/CK20 antibody panel to a cohort of MIBC patients treated with platinum-based neoadjuvant chemotherapy and found significantly higher rates of complete response in the basal (CK5/6+/CK20-) group (*p* = 0.037) [27]. Interestingly, an inverse correlation between CK5 and CK20 has been reported in Ta NMIBCs, the two markers being negatively and positively associated with high-grade disease, respectively [28]. In our study, the exceeding GATA3 expression rate accounts for the almost double proportion of cases labeled as luminal according to Scheme #2 (60.2% vs. 32.3%), as well as the lower number of basal cases (9.7% vs. 33.3%).

Although GATA3 and CK5/6 are considered quite specific luminal and basal markers, respectively, GATA3+/CK5/6+ double-positive (DP) cases have been described in approximately 43–48% of MIBC cases [26,27]. DN cases have been described in previous studies using the dual-antibody CK5/6/GATA3 classifier [22,28], accounting for 3–15% of cases. In our study, CK5/6-/GATA3- tumors were approximately 3%. Such DN and DP cases have been suggested to represent either transition forms between subtypes or separate subtypes with their own underlying molecular profile; in keeping with the latter hypothesis, the DP (CK5/6+/CK20+) subgroup described by Kim et al. showed a mixed luminal/basal gene expression signature, as well as a stronger immune signature gene expression [21]. The true meaning of DP and DN cases remains to be disclosed.

In our study, we did not observe consistent differences in terms of OS and CSS between the molecular subtypes obtained through surrogate IHC markers of any kind, in keeping with previous studies [16,24,29,30,31,32,33]. Serag-Eldien et al. [34] applied a CK5/6/GATA3 classifier to their cohort of 80 BCs of any grade and stage and reported a trend of better OS and PFS for luminal MIBCs, although no statistical significance was achieved, in line with our results. Conversely, in the study by Olkhov-Mitsel et al., basal (CK5/6+/GATA3-) MIBCs were independent predictors of worse disease-specific survival as compared to urothelial-like (CK5/6-/GATA3+/p16-) tumors (*p* = 0.033) [31]. In keeping with this, specimens enriched with a luminal (CK5/6- and/or CK14-/GATA3+) phenotype showed higher PFS (*p* = 0.032) in a cohort of MIBCs mostly treated with adjuvant cisplatin-based chemotherapy [30].

In our study, we found a significant association between the pattern of MP invasion and the basal, DN, and DP molecular subtypes according to Scheme #2, in that all 8 CK5/6+/GATA3-, all 3 CK5/6-/GATA3-, and 83% of CK5/6+/GATA3+ cases had a non-infiltrative invasion pattern. The pattern of MP invasion in MIBC has been investigated in previous studies. Jimenez et al. recognized three types of invasive patterns in their cohort of MIBC, namely, nodular, trabecular, and infiltrative, the latter being associated with shorter survival, though not significantly (*p* = 0.06) [35]. In a later study, Langner et al. applied the same classification scheme to a large cohort of upper tract UCs, reporting a consistent association between tumor stage (*p* < 0.001) and the infiltrative pattern (*p* < 0.001) with metastasis-free survival in a multivariate analysis [36]. Recently, Haghayeghi et al. subclassified MP invasion of their 43 pT2 MIBCs into 2 patterns, each showing mainly the features of nodular (pattern 1) and infiltrative (pattern 2) patterns by Jimenez et al., whereas trabecular morphology was seen in both patterns 1 and 2 [35]. Pattern 2 invasion was consistently associated with a higher (pT2b) stage (*p* = 0.02) and also with aggressive features, such as LVI, PNI, and the presence of nodal metastases, though not significantly. Conversely, pattern 1 showed more frequent expression of luminal markers, such as GATA3 (*p* = 0.004) and HER2 (*p* = 0.04).

### Study Limitations

Our study had some limitations. First, this was a retrospective, single-center study with a relatively small, though homogeneous, sample size. Second, we did not perform mRNA profiling to confirm molecular subtypes since high concordance rates between mRNA-based taxonomy and IHC classification systems have already been demonstrated in several large cohorts of MIBC [37]. Third, despite its many advantages, IHC still needs to be improved in this setting; the ongoing advancement in digital pathology, resulting in the widespread implementation of image analysis tools [38], opens up promising prospects for improving this method through the development and validation of standardized interpretation and/or quantification criteria. Finally, we did not assess the predictive role of such markers, unlike other studies [39], since we instead focused on their prognostic role.

## 4. Materials and Methods

### 4.1. Case Selection

After Institutional Review Board approval, we retrospectively searched our database in order to identify all patients affected by MIBC who underwent radical cystectomy (RC) at the Urology Department of the University Hospital of Foggia, Italy between 2015 and 2020. Only cases with sufficient material available for immunohistochemistry, lacking autolysis artifact, were selected. Eventually, 93 cases were included in the present study.

### 4.2. Histological Evaluation

Hematoxylin- and eosin-stained sections were reviewed to assess the diagnosis and stage according to the 2022 World Health Organization classification [17] and the 2017 TNM staging system [18] by two dedicated uropathologists (FS and ST) blinded to clinical outcomes. The histological subtypes of urothelial carcinoma (UC), including pure and variant histology (VH), namely, squamous, micropapillary, plasmacytoid, and sarcomatoid, were identified [17], along with the presence/absence of papillary architecture, cytologic anaplasia, urothelial carcinoma in situ (CIS), lymphovascular and/or perineural invasion, tumor size, and necrosis. The deep invasive component in the detrusor muscle was assessed in each case and subclassified into an infiltrative pattern (IP, with narrow cords and/or small aggregates and/or single cells infiltrating and/or dissecting into the muscular bundles) and a trabecular–nodular pattern (TNP, with broad trabecular and/or large aggregates of tumor cells) [19].

### 4.3. Immunohistochemistry (IHC)

Formalin-fixed paraffin-embedded blocks were retrieved and 4 μm tissue sections were obtained. After deparaffinization and rehydration, tissue sections were subjected to antigen retrieval and primary antibody incubation with 5 different primary antibodies against CK5/6 (clone D5/16B4, rabbit monoclonal); CK7 (SP52, rabbit monoclonal); CK20 (SP33, rabbit monoclonal); keratin (34βE12, mouse monoclonal); and GATA3 (L50-823, mouse monoclonal), with appropriate positive and negative controls. Primary antibody was omitted for negative controls. All immunostaining was performed on a Benchmark XT automated stainer (Ventana Medical Systems, Tucson, AZ, USA) with primary antibody incubations of around 30 min at room temperature.

The staining intensity, graded as 0 (negative), 1 (weak), 2 (moderate), and 3 (strong), and percentage of immunoreactive cells (0–100%) were assessed for each antibody. The semi-quantitatively combined H-score, ranging from 0 to 300, was obtained by multiplying both. Cases yielding a final H-score as high as ≥150 were classified as positive [20].

### 4.4. Determination of Molecular Subtypes

Two scoring schemes based on the immunohistochemical phenotypes were used to establish the molecular subtypes [21,22]; the coordinate expression of CK5/6 and CK20 (Scheme #1) and CK5/6 and GATA3 (Scheme #2) was assessed, with CK5/6 and CK20-GATA3 used as basal and luminal markers, respectively.

### 4.5. Survival and Follow-Up Data

All patients were followed up every three months after RC. Survival data were collected from medical records in July 2021, gathering dates and causes of deaths. Median survivor follow-up length was 28 (IQR: 16, 38) months.

### 4.6. Statistical Analysis

Descriptive statistics are reported for the overall population and stratifying according to each marker expression. Continuous variables are reported as median and interquartile range and tested by the Mann–Whitney U-test, whereas categorical variables are reported as rates and tested by Fisher’s exact test or the chi-square test, as appropriate.

Overall survival and cancer-specific survival were estimated non-parametrically using the Kaplan–Meier method, with differences among groups being tested for significance using the Log-rank test. Univariable semi-parametric Cox regression analyses were used to evaluate the association between pathological and immunohistochemical parameters with overall survival and cancer-specific survival.

Statistical analyses were performed using Stata-SE 14 (StataCorp LP, College Station, TX, USA). All tests were 2-sided with a significance level set at *p* < 0.05.

## 5. Conclusions

In conclusion, comprehensive pathological evaluation of morphological features, including the pattern of MP invasion, along with the identification of BC subtypes, may be used to refine the diagnosis of MIBC [40]. The use of proper antibody panels as IHC surrogates, such as the combined expression of CK5/6 and GATA3, represents a more cost-effective and efficient method than mRNA profiling in routine practice. Nevertheless, prospective validation of these preliminary findings in large datasets is advisable prior to clinical implementation.

## Figures and Tables

**Table 1:** Clinicopathological characteristics of our cohort.

Variables	
**Age, years**	74 (69–78)
**Gender**	
Female	14 (15.1%)
Male	79 (84.9%)
**Treatment**	
TURBT	12 (12.9%)
RC	81 (87.1%)
**Papillary morphology**	
Absent	73 (90.1%)
Present	8 (9.9%)
**Histological subtypes**	
Presence of variant histology	24 (29.6%)
Conventional UC	57 (70.4%)
**Anaplasia**	
Absent	66 (81.5%)
Present	15 (18.5%)
**Carcinoma in situ (CIS)**	
Absent	64 (79.0%)
Present	17 (21.0%)
**pT stage**	
2	14 (17.3%)
3	36 (44.4%)
4	31 (38.3%)
**pN stage**	
0	50 (53.8%)
1	43 (46.2%)
**AJCC stage**	
II	9 (11.1%)
IIIA	45 (55.6%)
IIIB	27 (33.3%)
**Lymphovascular invasion (LVI)**	
Absent	42 (51.9%)
Present	39 (48.1%)
**Perineural invasion (PNI)**	
Absent	50 (61.7%)
Present	31 (38.3%)
**Pattern of muscularis propria invasion**	
Infiltrative	25 (26.9%)
Non-infiltrative	68 (73.1%)

**Table 2:** CK5/6, CK7, CK20, CK34βE12, and GATA3 expression in MIBCs regarding different clinicopathological features. *p*-value < 0.05 are highlighted in bold.

	CK5/6 (*n* = 31)	*p*-Value	CK7 (*n* = 65)	*p*-Value	CK20 (*n* = 26)	*p*-Value	CK34BE12 (*n* = 65)	*p*-Value	GATA3 (*n* = 70)	*p*-Value
**Age**	73 (69–77)	0.9	74 (69–78)	0.7	74 (68–76)	0.4	73 (69–78)	0.7	74 (69–78)	0.6
**Gender**										
Female	8 (26%)	**0.028**	10 (15%)	0.8	3 (12%)	0.6	10 (15%)	0.8	8 (11%)	**0.030**
Male	23 (74%)		55 (85%)		23 (88%)		55 (85%)		62 (89%)	
**Papillary morphology**										
Present	30 (97%)	0.11	57 (88%)	0.14	22 (85%)	0.3	59 (91%)	0.7	62 (89%)	0.2
Absent	1 (3%)		8 (12%)		4 (15%)		6 (9%)		8 (11%)	
**Histological subtypes**										
Presence of variant histology	11 (35%)	0.4	16 (25%)	**0.046**	7 (27%)	0.7	20 (31%)	0.7	19 (27%)	0.2
Conventional UC	20 (65%)		49 (75%)		19 (73%)		45 (69%)		51 (73%)	
**Anaplasia**										
Absent	24 (77%)	0.5	53 (82%)	1	20 (77%)	0.5	51 (78%)	0.2	58 (83%)	0.4
Present	7 (23%)		12 (18%)		6 (23%)		14 (22%)		12 (17%)	
**Carcinoma in situ (CIS)**										
Absent	27 (87%)	0.2	51 (78%)	0.8	20 (77%)	0.8	54 (83%)	0.070	55 (79%)	0.8
Present	4 (13%)		14 (22%)		6 (23%)		11 (17%)		15 (21%)	
**pT stage**										
2	4 (13%)	0.5	12 (18%)	0.6	3 (12%)	0.14	10 (15%)	0.4	12 (17%)	0.4
3	16 (52%)		27 (42%)		9 (35%)		28 (43%)		33 (47%)	
4	11 (35%)		26 (40%)		14 (54%)		27 (42%)		25 (36%)	
**pN stage**										
0	16 (52%)	0.5	32 (49%)	0.4	11 (42%)	0.6	30 (46%)	0.8	33 (47%)	0.9
1	15 (48%)		33 (51%)		15 (58%)		35 (54%)		37 (53%)	
**AJCC stage**										
II	4 (13%)	0.5	8 (12%)	0.5	2 (8%)	0.5	6 (9%)	0.6	7 (10%)	0.7
IIIA	19 (61%)		34 (52%)		13 (50%)		37 (57%)		40 (57%)	
IIIB	8 (26%)		23 (35%)		11 (42%)		22 (34%)		23 (33%)	
**Lymphovascular invasion (LVI)**										
Absent	20 (65%)	0.072	33 (51%)	0.7	10 (38%)	0.10	36 (55%)	0.2	37 (53%)	0.6
Present	11 (35%)		32 (49%)		16 (62%)		29 (45%)		33 (47%)	
**Perineural invasion (PNI)**										
Absent	19 (61%)	0.9	38 (58%)	0.2	15 (58%)	0.6	41 (63%)	0.6	41 (59%)	0.14
Present	12 (39%)		27 (42%)		11 (42%)		24 (37%)		29 (41%)	
**Pattern of MP invasion**										
Infiltrative	4 (13%)	**0.009**	21 (32%)	0.3	10 (38%)	0.2	16 (25%)	**0.046**	24 (34%)	**0.021**
Non-infiltrative	27 (87%)		44 (68%)		16 (62%)		49 (75%)		46 (66%)	

**Table 3:** Association between molecular subtypes obtained through Scheme #1 and clinicopathological variables.

	Basal (*n* = 31)	Luminal (*n* = 30)	Double Positive (*n* = 3)	Double Negative (*n* = 29)	*p*-Value
**Age**	73 (70–78)	74 (69–77)	69 (59–90)	75 (69–81)	0.9
**Gender**					
Female	7 (23%)	3 (10%)	1 (33%)	3 (10%)	0.4
Male	24 (77%)	27 (90%)	2 (67%)	26 (90%)	
**Papillary morphology**					
Absent	28 (97%)	20 (83%)	2 (100%)	23 (88%)	0.4
Present	1 (3%)	4 (17%)	0 (0%)	3 (12%)	
**Histological subtypes**					
Presence of variant histology	10 (34%)	6 (25%)	1 (50%)	7 (27%)	0.8
Conventional UC	19 (66%)	18 (75%)	1 (50%)	19 (73%)	
**Anaplasia**					
Absent	23 (79%)	19 (79%)	1 (50%)	23 (88%)	0.5
Present	6 (21%)	5 (21%)	1 (50%)	3 (12%)	
**Carcinoma in situ (CIS)**					
Absent	25 (86%)	18 (75%)	2 (100%)	19 (73%)	0.5
Present	4 (14%)	6 (25%)	0 (0%)	7 (27%)	
**pT stage**					
2	4 (14%)	3 (12%)	0 (0%)	7 (27%)	0.4
3	15 (52%)	8 (33%)	1 (50%)	12 (46%)	
4	10 (34%)	13 (54%)	1 (50%)	7 (27%)	
**pN stage**					
0	17 (55%)	16 (53%)	2 (67%)	15 (52%)	1
1	14 (45%)	14 (47%)	1 (33%)	14 (48%)	
**AJCC stage**					
II	4 (14%)	2 (8%)	0 (0%)	3 (12%)	0.9
IIIA	18 (62%)	12 (50%)	1 (50%)	14 (54%)	
IIIB	7 (24%)	10 (42%)	1 (50%)	9 (35%)	
**Lymphovascular invasion (LVI)**					
Absent	19 (66%)	9 (38%)	1 (50%)	13 (50%)	0.2
Present	10 (34%)	15 (62%)	1 (50%)	13 (50%)	
**Perineural invasion (PNI)**					
Absent	18 (62%)	14 (58%)	1 (50%)	17 (65%)	0.9
Present	11 (38%)	10 (42%)	1 (50%)	9 (35%)	
**Pattern of MP invasion**					
Infiltrative	4 (13%)	11 (37%)	0 (0%)	10 (34%)	0.090
Non-infiltrative	27 (87%)	19 (63%)	3 (100%)	19 (66%)	

**Table 4:** Association between molecular subtypes obtained through Scheme #2 and clinicopathological variables. *p*-value < 0.05 are highlighted in bold.

	Luminal (*n* = 56)	Basal (*n* = 9)	Double Positive (*n* = 25)	Double Negative (*n* = 3)	*p*-Value
**Age**	74 (69, 80)	73 (72, 74)	74 (69, 78)	71 (69, 78)	1
**Gender**					
Female	5 (9%)	3 (33%)	5 (20%)	1 (33%)	0.2
Male	51 (91%)	6 (67%)	20 (80%)	2 (67%)	
**Papillary morphology**					
Absent	40 (85%)	8 (100%)	22 (96%)	3 (100%)	0.3
Present	7 (15%)	0 (0%)	1 (4%)	0 (0%)	
**Histological subtypes**					
Presence of variant histology	13 (28%)	5 (62%)	6 (26%)	0 (0%)	0.13
Conventional UC	34 (72%)	3 (38%)	17 (74%)	3 (100%)	
**Anaplasia**					
Absent	39 (83%)	5 (62%)	19 (83%)	3 (100%)	0.4
Present	8 (17%)	3 (38%)	4 (17%)	0 (0%)	
**Carcinoma in situ (CIS)**					
Absent	36 (77%)	8 (100%)	19 (83%)	1 (33%)	0.10
Present	11 (23%)	0 (0%)	4 (17%)	2 (67%)	
**pT stage**					
2	10 (21%)	2 (25%)	2 (9%)	0 (0%)	0.4
3	19 (40%)	2 (25%)	14 (61%)	1 (33%)	
4	18 (38%)	4 (50%)	7 (30%)	2 (67%)	
**pN stage**					
0	30 (54%)	5 (56%)	14 (56%)	1 (33%)	0.9
1	26 (46%)	4 (44%)	11 (44%)	2 (67%)	
**AJCC stage**					
II	5 (11%)	2 (25%)	2 (9%)	0 (0%)	0.6
IIIA	25 (53%)	4 (50%)	15 (65%)	1 (33%)	
IIIB	17 (36%)	2 (25%)	6 (26%)	2 (67%)	
**Lymphovascular invasion (LVI)**					
Absent	22 (47%)	5 (62%)	15 (65%)	0 (0%)	0.13
Present	25 (53%)	3 (38%)	8 (35%)	3 (100%)	
**Perineural invasion (PNI)**					
Absent	28 (60%)	6 (75%)	13 (57%)	3 (100%)	0.4
Present	19 (40%)	2 (25%)	10 (43%)	0 (0%)	
**Pattern of MP invasion**					
Infiltrative	21 (38%)	0 (0%)	4 (16%)	0 (0%)	**0.028**
Non-infiltrative	35 (62%)	9 (100%)	21 (84%)	3 (100%)	

## Data Availability

The data presented in this study are available on request from the corresponding author.
